# Design, synthesis and molecular modelling studies of some pyrazole derivatives as carbonic anhydrase inhibitors

**DOI:** 10.1080/14756366.2019.1695791

**Published:** 2019-12-04

**Authors:** Yazgı Dizdaroglu, Canan Albay, Tayfun Arslan, Abdulilah Ece, Emir A. Turkoglu, Asiye Efe, Murat Senturk, Claudiu T. Supuran, Deniz Ekinci

**Affiliations:** a Faculty of Science and Arts, Giresun University, Giresun, Turkey; b Technical Sciences Vocational School, Giresun University, Giresun, Turkey; c Faculty of Pharmacy, Biruni University, Istanbul, Turkey; d Faculty of Pharmacy, University of Health Sciences, Istanbul, Turkey; e Faculty of Pharmacy, Agri Ibrahim Cecen University, Agri, Turkey; f Neurofarba Department, University of Florence, Firenze, Italy; g Ondokuz Mayis University, Faculty of Agriculture, Department of Agricultural Biotechnology, Samsun, Turkey

**Keywords:** Pyrazole, carbonic anhydrase, molecular docking, ADME

## Abstract

In this study, newly synthesised compounds **6**, **8**, **10** and other compounds (**1–5**, **7** and **9**) and their inhibitory properties against the human isoforms hCA I and hCA II were reported for the first time. Compounds **1–10** showed effective inhibition profiles with *K*_I_ values in the range of 5.13–16.9 nM for hCA I and of 11.77–67.39 nM against hCA II, respectively. Molecular docking studies were also performed with Glide XP to get insight into the inhibitory activity and to evaluate the binding modes of the synthesised compounds to hCA I and II. More rigorous binding energy calculations using MM-GBSA protocol which agreed well with observed activities were then performed to improve the docking scores. Results of *in silico* calculations showed that all compounds obey drug likeness properties. The new compounds reported here might be promising lead compounds for the development of new potent inhibitors as alternatives to classical hCA inhibitors.

## 1. Introduction

Carbonic anhydrases (CAs, EC 4.2.1.1) are one of the metalloenzymes catalysing the hydration process of CO_2_ to HCO_3_^−^ and H^+^. All living organisms contain CAs encoded by six phylogenetically gene families.^1^^,^^2^ Fifteen CA isoenzymes belonging to α-CA gene family have been characterised in human-beings. Some human CA (hCA) isoenzymes are cytosolic isoforms (hCA I, II, III, VII and XIII), some isoenzymes are membrane-bound isoforms (hCA IV, IX, XII and XIV), both hCA VA and VB are mitochondrial isoforms and hCA VI isoform is involved in saliva. Three hCA isoenzymes (CA VIII, X and XI) are characterised as acatalytic protein forms. Inhibition and activation studies on the catalytic activity of CAs are crucial for the treatment of numerous clinically important diseases.^2^^,^^3^ The inhibitors of CA isoenzymes (e.g., CA I and CA II) are used to design new class of drugs for epilepsy and glaucoma. Therefore, new CA inhibitors have been required to develop as therapeutic agents.^2–4^ Several groups have studied the inhibition of hCAs with anions,^4^ catecholamines,^5^ thiourea derivatives,^6^ uracil derivatives,^7^ bromophenols^8^ and sulphonamides.^9^ In addition, pyrazoles and chalcones have also been studied to inhibit hCAs as well.^10^

Heterocyclic compounds have a vital role in medicine, pharmacy and agriculture.^11^ Pyrazoles possess various important bio-medical features.^12^ This type of derivatives exhibits several therapeutic activities such as insecticidal,^13^ acaricidal,^14^ anticonvulsant^15^ antidepressant,^16^ antiulcer, and anticancer features.^17^ So far to date, many chalcone derivatives have been synthesised and their biological activities examined.^18^ Several investigations have shown that chalcones possess important pharmacological characteristics including antitumor, anti-inflammatory, antifungal and antioxidant properties.^19^ The development of effective CA inhibitors is limited by the lack of selectivity which could lead to serious side effects.^2^ Hence, it has been of interest to us to develop not only potent hCA inhibitors but also with a promising selectivity for a specific isoform. We have previously carried out synthesis of various phenols and methoxyphenols in addition to derivatives of some natural products which possess different structures.^20^ Some of our recently synthesised compounds were found to inhibit CAs in the milimolar to low nanomolar ranges.^21^ In the current study, we focussed on the synthesis and inhibitory effects of some pyrazole derivatives against hCA I and II isoforms. Computational studies were also used to enlighten their activities based on the binding interactions with the target enzymes and their calculated molecular properties.

## 2. Methods and materials

### 2.1. Chemistry

^1^H and 13 C spectra were recorded on Bruker Ascend 400 (100)–MHz spectrometers and chemical shifts were reported (λ) relative to Me_4_Si as internal standard. The elemental analyses were performed on a Costech ESC 4010 instrument. The IR spectra were determined using a Perkin Elmer 1600 Fourier Transform-infrared (FT-IR) spectrophotometer on a KBr disc. Melting points were determined by using a Barnstead electrothermal 9200 series digital apparatus. Absorption spectra were recorded on a Shimadzu UV-1800 spectrophotometer. CA esterase activity was determined according to Verpoorte *et al*.^22^

### 2.2. General methods for the synthesis of chalcones (1–5)

An aqueous solution of NaOH (60%, 10 ml) was added into the ethanol (6 ml) solution of substituted carbaldehyde (20.0 mmol) and a suitable acetophenone (20.0 mmol). The mixture was stirred for a day at room temperature and it was then poured on ice-water. The mixture was neutralised using 6 M hydrochloric acid. The yellow precipitate obtained was filtered and crystallized from ethanol-water. (E)-1-(4-aminophenyl)-3-(3,4,5-trimethoxyphenyl)prop-2-en-1-one (1), (E)-1-(4-bromophenyl)-3-(3,4,5-trimethoxyphenyl)prop-2-en-1-one (2), (E)-1-p-tolyl-3-(3,4,5-trimethoxyphenyl)prop-2-en-1-one (3), (E)-1-(4-aminophenyl)-3-(3,4-dimethoxyphenyl)prop-2-en-1-one (4), (E)-3-(4-(dimethylamino)phenyl)-1-(4-hydroxyphenyl)prop-2-en-1-one (5) were synthesised according to the literature,^23^ respectively.

#### 2.2.1. General methods for the synthesis of pyrazoles (6–10)

A mixture of (0.007 mol) chalcone and (0.014 mol) thiosemicarbazide were refluxed in ethanol (15 ml) while stirring vigorously. After complete dissolution of the reactants, a solution of (0.014 mol) of KOH in ethanol (15 ml) was added dropwise. The solution was refluxed for another 18 h, allowed to warm at room temperature and then stirred for 4 h. The crude product was refrigerated overnight. The precipitate formed was filtered off and crystallized from ethanol twice yielding yellow crystals.

#### 2.2.2. 3-(4-Aminophenyl)-5-(3,4,5-trimethoxyphenyl)-4,5-dihydro-1H-pyrazole-1-carbothioamide (6)

IR (ATR), ν/cm^−1^: 3439, 3331, 1591, 1342. ^1^H-NMR (DMSO-d_6_), (δ:ppm): 3.10 (1H, dd, J = 3.2 and 3.2 Hz), 3.62 (3H, s), 3.70 (6H, s), 3.86 (1H, dd, J = 13.6 and 10.8 Hz), 5.80 (1H, dd, J = 3.2 and 2.8 Hz), 6.40 (2H, s), 6.56 (2H, d, J = 8.8 Hz), 7.53 (2H, d, J = 8.4 Hz). ^13 ^C-NMR (DMSO-d_6_), (δ:ppm): 175.80, 156.52, 153.35, 151.91, 139.31, 136.82, 129.21, 118.08, 113.71, 103.07, 62.93, 60.38, 56.59, 42.95. Anal. calcd. for: C_19_H_22_N_4_O_3_S: C, 59.05; H, 5.74; N, 14.50; Found: C, 59.07; H, 5.71; N, 14.48.

#### 2.2.3. 3-(4-Bromophenyl)-5-(3,4,5-trimethoxyphenyl)-4,5-dihydro-1H-pyrazole-1-carbothioamide (7)

IR (ATR), ν/cm^−1^: 3439, 3263, 1587, 1334. ^1^H-NMR (DMSO-d_6_), (δ:ppm): 3.21 (1H, dd, J = 3.6 and 3.2 Hz), 3.64 (3H, s), 3.71 (6H, s), 3.90 (1H, dd, J = 12.6 and 12.0 Hz), 5.89 (1H, dd, J = 2.8 and 2.8 Hz), 6.43 (2H, s), 7.65 (2H, d, J = 8.4 Hz), 7.82 (2H, d, J = 8.4 Hz). ^13 ^C-NMR (DMSO-d_6_), (δ:ppm): 176.20, 154.52, 153.80, 139.64, 136.90, 132.07, 130.42, 129.54, 124.46, 104.37, 63.57, 59.99, 55.98, 42.75. Anal. calcd. for: C_19_H_20_BrN_3_O_3_S: C, 50.67; H, 4.48; N, 9.33; Found: C, 50.65; H, 4.47; N, 9.29.

#### 2.2.4. 3-p-Tolyl-5-(3,4,5-trimethoxyphenyl)-4,5-dihydro-1H-pyrazole-1-carbothioamide (8)

IR (ATR), ν/cm^−1^: 3433, 3263, 1580, 1338. ^1^H-NMR (DMSO-d_6_), (δ:ppm): 2.35 (3H, s), 3.20 (1H, dd, J = 3.6 and 3.2 Hz), 3.64 (3H, s), 3.71 (6H, s), 3.92 (1H, dd, J = 12.0 and 11.0 Hz), 5.88 (1H, dd, J = 3.2 and 2.8 Hz), 6.43 (2H, s), 7.27 (2H, d, J = 4 Hz), 7.76 (2H, d, J = 4 Hz). ^13 ^C-NMR (DMSO-d_6_), (δ:ppm): 176.76, 155.73, 153.40, 141.02, 139.18, 136.88, 129.74, 128.59, 127.59, 103.05, 63.34, 60.38, 56.63, 39.68, 21.50. Anal. calcd. for: C_20_H_23_N_3_O_3_S: C, 62.32; H, 6.01; N, 10.90; Found: C, 62.31; H, 6.02; N, 10.89.

#### 2.2.5. 3-(4-Aminophenyl)-5-(3,4-dimethoxyphenyl)-4,5-dihydro-1H-pyrazole-1-carbothioamide (9)

IR (ATR), ν/cm^−1^: 3443, 3304, 1576, 1357. ^1^H-NMR (DMSO-d_6_), (δ:ppm): 3.06 (1H, dd, J = 2.8 and 2.8 Hz), 3.70 (3H, s), 3.71 (3H, s), 3.82 (1H, dd, J = 7.2 and 6 Hz), 5.73 (1H, s), 5.81 (1H, dd, J = 2.8 and 2.4 Hz), 6.57 (2H, d, J = 8.8 Hz), 6.77 (1H, d, J = 2), 6.85 (1H, d, J = 8.4), 7.53 (2H, d, J = 8.8 Hz). ^13 ^C-NMR (DMSO-d_6_), (δ:ppm): 175.51, 156.48, 151.87, 149.08, 148.16, 136.04, 130.61, 129.17, 114.78, 113.73, 112.29, 110.21, 65.92, 55.99, 55.93, 42.93. Anal. calcd. for: C_18_H_20_N_4_O_2_S: C, 60.65; H, 5.66; N, 15.72; Found: C, 60.64; H, 5.63; N, 15.72.

#### 2.2.6. 5-(4-(Dimethylamino)phenyl)-3-(4-hydroxyphenyl)-4,5-dihydro-1H-pyrazole-1-carbothioamide (10)

IR (ATR), ν/cm^−1^: 3430, 3260, 1580, 1346. ^1^H-NMR (DMSO-d_6_), (δ:ppm): 2.99 (6H, s), 3.06 (1H, dd, J = 2.8 and 2.0 Hz), 3.72 (1H, dd, J = 10.8 and 11.2 Hz), 5.78 (1H, dd, J = 2.4 and 2.4 Hz), 6.65 (2H, d, J = 8.8 Hz), 6.83 (2H, d, J = 8.8 Hz), 6.95 (2H, d, J = 8.4 Hz), 7.71 (2H, d, J = 8.4 Hz), 7.82 (br, –NH_2_), 11.20 (br, –OH). ^13 ^C-NMR (DMSO-d_6_), (δ:ppm): 175.75, 160.32, 156.50, 151.91, 130.31, 129.21, 127.15, 119.01, 113.95, 63.81, 42.05, 41.78. Anal. calcd. for: C_18_H_20_N_4_OS: C, 63.50; H, 5.92; N, 16.46; Found: C, 63.54; H, 5.88; N, 16.44.

### 2.3. Biological activity

#### 2.3.1. Inhibition studies of carbonic anhydrase I and II isoforms

Enzyme activity was determined spectrophotometrically by following the change in absorbance at 348 nm of 4-nitrophenylacetate to 4-nitrophenolate over a period of 3 min at 25 °C.^21^ The enzymatic reaction contained 1.4 ml 0.05 M Tris-SO_4_ buffer (pH 7.4), 1 ml 3 mM 4-nitrophenylacetate, 0.5 ml H_2_O and 0.1 ml enzyme solution, in a total volume of 3.0 ml.^24^ Inhibitory effects of compounds **1**–**10** were compared with acetazolamide (**AZA**). Different inhibitor concentrations were used and all compounds were tested in triplicate at each concentration used. Control cuvette activity was acknowledged as 100% in the absence of inhibitor. An Activity% – [Inhibitor] graph was drawn for each inhibitor.^25^ The curve-fitting algorithm allowed for obtaining the IC_50_ values, working at the lowest concentration of substrate of 0.15 mM, from which K_i_ values were calculated.^20^^,^^21^ The catalytic activity of these enzymes was calculated from Lineweaver-Burk plots, as reported previously, and represent the mean from at least three different determinations. The hCA I and II isoenzymes used here were purified from human blood as previously described.^21^

### 2.4. Computational section

#### 2.4.1. Ligand and protein preparation

As a crucial step to meet minimum requirements for further computational calculations, all the studied ligands and target proteins were prepared. LigPrep tool^26^ interfaced with Maestro module of Schrödinger^26^ suite was used for the ligand preparation. The 3 D structures including all possible tautomers and ionisation states at pH 7.0 ± 2.0 of all the ligands **1–10** and the reference compound **AZA** were generated and geometrically minimised using optimised potential liquid simulations (OPLS3) force field.^27^

Schrödinger’s multi-step Protein Preparation Wizard PrepWizard)^28^ were used for the protein preparations. As an initial step, high-resolution protein crystal structures of CA I and II (PDB Ids: 2NMX and 3HS4, respectively), both in complexed with a native ligand, were retrieved from RCSB Protein Data Bank. Charges and bond orders were assigned, hydrogens were added to the heavy atoms, all water molecules and heteroatoms were then removed keeping the native ligands and zinc metals in the active site. The final structures were optimised and finally minimised using OPLS3 force field to avoid steric clashes between the atoms.

#### 2.4.2. Molecular docking

A grid representing the binding pocket was generated using the centroid of co-crystallized native ligands. Default settings were kept in each case. Glide XP (extra precision)^29^ module of Schrödinger Suite was used to dock the synthesised compounds into the active site of the crystal structures. The rescoring was performed to calculate and improved binding energy calculations with Prime’s Molecular Mechanics‐Generalized Born Surface Area (MM‐GBSA) protocol using VSGB solvation model.^26^^,^^30^

#### 2.4.3. Calculation of physicochemical and ADME properties

QikProp^26^ module of Schrodinger was used to calculate some commonly used molecular descriptors such as dipole moment, logarithm of octanol-water partition coefficient (QPlogPo/w), percent human oral absorption, polar surface area (PSA) and violations to the Lipinski’s rule of five.^30^

## 3. Results and discussion

### 3.1. Chemistry

**Figure 1.**
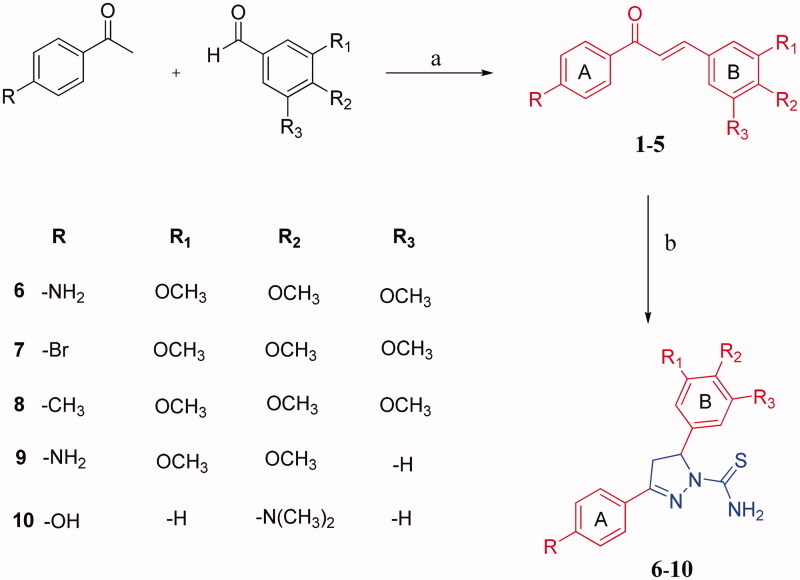
(a) 60% Aq NaOH, EtOH, 24 h; (b) Thiosemicarbazide, KOH, EtOH, reflux 18 h.

**Figure 2.**
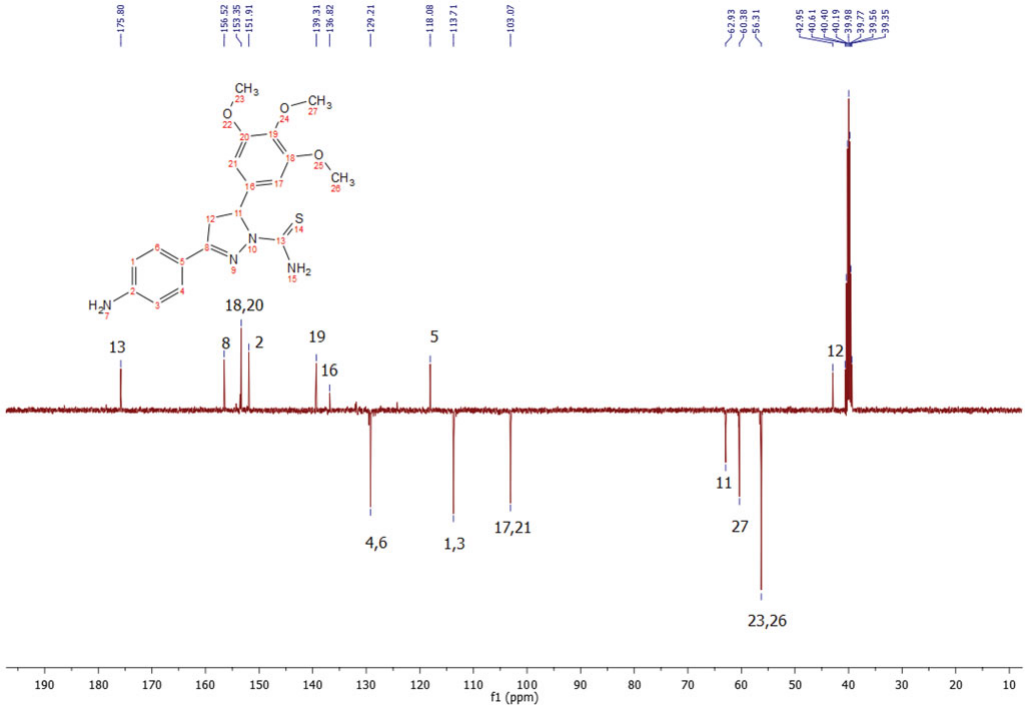
^13 ^C NMR spectrum of compound 6.

The new 4,5-dihydro-*1H*-pyrazole-1-carbothioamide derivatives (**6–10**) were prepared from the chalcones **1**and **5** according to the reactions outlined in Figure 1. Initially, chalcones (**1–5**) were prepared through Claisen-Schmidt condensation, which is the most important reaction in the formation of 1,3-diphenyl-2-propene-1-ones (chalcones), of various benzaldehydes with 4-amino, bromo, methyl and hydroxyl acetophenones. The reaction was carried out in 60% sodium hydroxide: ethanol for 24 h as stated in previous works.^23^ Then, new 1-thiocarbamoyl-3,5-diaryl-4,5-dihydro-(1*H*)-pyrazole derivatives (**6–10**) were obtained by cyclisation of chalcones (**1–5**) with two equivalents of thiosemicarbazide with sodium hydroxide presence in ethanol. Compound **7**–**9** was reported in a previous study.^23^ The synthesis of the compounds **6, 8** and **10** is being reported for the first time in this work. At the end of the synthesis, the crude product was purified by recrystallization two times from EtOH: H_2_O to obtain yellow crystals. The structures of all the compounds were confirmed by spectral FT-IR, ^1^H and 13 C NMR and elemental analyses. ^1^H NMR spectra of the title compounds were consistent with expected resonance signals in terms of chemical shifts and integrations. All the 13 C NMR findings confirmed the structures proposed. Selected 13 C NMR spectrum are given in Figure 2.

### 3.2. Biological evaluation

The inhibitory effects of all the synthesised compounds were investigated for the first time against hCA isozymes: hCA I and hCA II. Human CA I and II isozymes were purified by one step chromatography technique and the activity of the effluents was determined by the hydratase method, using CO_2_ as substrate and further kinetic studies were performed using the esterase activity method, using 4-nitrophenyl acetate (NPA) as substrate.^21^ Inhibition characteristics of five pyrazoles, five chalcones and various reference compounds are given in Table 1. It is clear from the results that all molecules were found to act as low-nanomolar hCA I-II inhibitors. According to the experimental findings, all chalcone and new pyrazole compounds used in this study had better inhibition constants than the clinically used inhibitor acetazolamide (AZA) and other widely used inhibitors (**11**, **12**, **13**) and also comparable IC_50_ values with AZA. However, they possessed different selectivity against hCAI-II. The new compounds **6** and **10** with the IC_50_ values ranging between 23.87 and 24.37 nM showed promising powerful inhibitory profiles compared to the standard drug AZA and they all had comparable IC_50_ values against hCA I. The amino or hydroxyl substituents on phenyl ring could easily be predicted to be involved in making hydrogen bonds with the active site as observed in classical hCA I sulphonamide inhibitors. Changing the hydrophilic substituents, –NH_2_ (**6**) or –OH (**10**) with hydrophilic substituents, –Br (**7**) or –CH_3_ (**8**) on ring A, have negligible effect on the observed activity. It is clear that the lack of moieties which can make favourable hydrogen bonding are being compensated with hydrophobic groups that also enhance affinity by hydrophobic interactions. Furthermore, replacing methoxy substituents with *N,N*-dimethylamino substituent (**10**) does not seem to have much effect on the activity. It can be concluded that methyl groups in both cases have favourable contacts with hydrophobic sites of the active region. Oxygen atoms in metoxy groups could have extra interactions with the hydrophilic regions. However, methoxy substituents on ring B have large impact on the activity towards hCA II. The IC_50_ value diminishes more than two-folds when trimethoxy phenyl is being replaced with N,N-dimethy aniline (**10**) which results in a promising selectivity profile for this compound. As a result of those observations, we found it necessary to carry out *in silico* studies.

**Table 1.** The data of hCA I and II inhibition with compounds **1–10** and **AZA**.

			*K_i_* (nM)^a^	
Compound	Yield (%)	M.p. (°C)	hCA I	hCA II
**1**	76	159–161	10.17	33.75
**2**	93	130–132	8.03	17.09
**3**	90	106–108	16.90	67.39
**4**	92	132–134	9.71	21.50
**5**	85	122–124	8.28	11.77
**6**	65	121–123	12.19	16.94
**7**	62	119–121	12.57	18.45
**8**	60	102–104	10.99	26.09
**9**	35	175–177	5.13	20.45
**10**	52	180–182	11.94	41.31
**AZA**			250^b^	12^b^

^a^Mean from at least three determinations. Errors in the range of 3% of the reported value (data not shown). ^b^From Ref. 31.

### 3.3. Computational study

**Figure 3.**
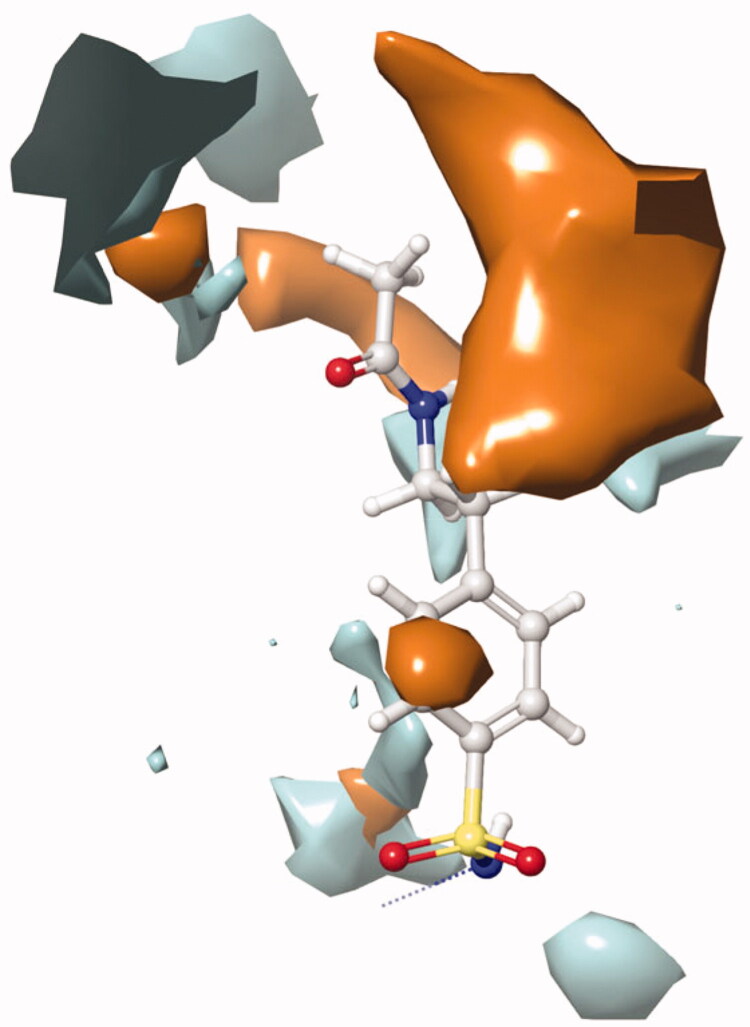
A representative hydrophobic/philic surfaces of the active site of hCA I complexed with a native ligand. (PDB ID: 2NMX; hydrophilic surfaces cyan colour; hydrophobic surfaces: orange colour).

**Figure 4.**
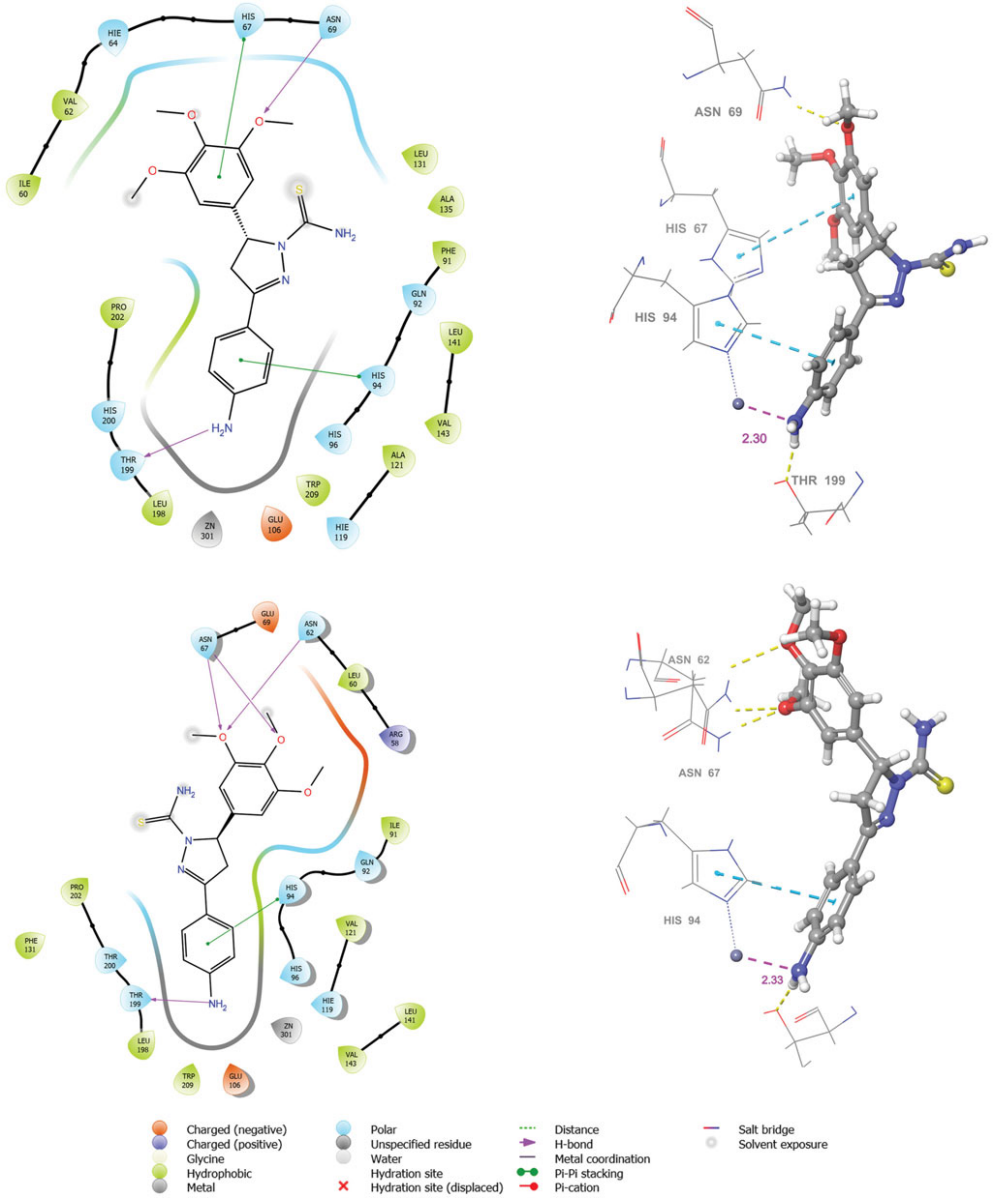
The 2 D and 3 D ligand interaction diagrams of hCA I-**6 (*R*)** (top) and hCA II-**6 (*R*)** (bottom) complexes obtained from prime MM-GBSA using Glide XP docked poses (In 3 D representation, hydrogen bonds are shown with yellow dashed lines, π-π interactions are shown with cyan colour. The distance between Zinc metal and interacting atom is shown with pink colour).

**Figure 5.**
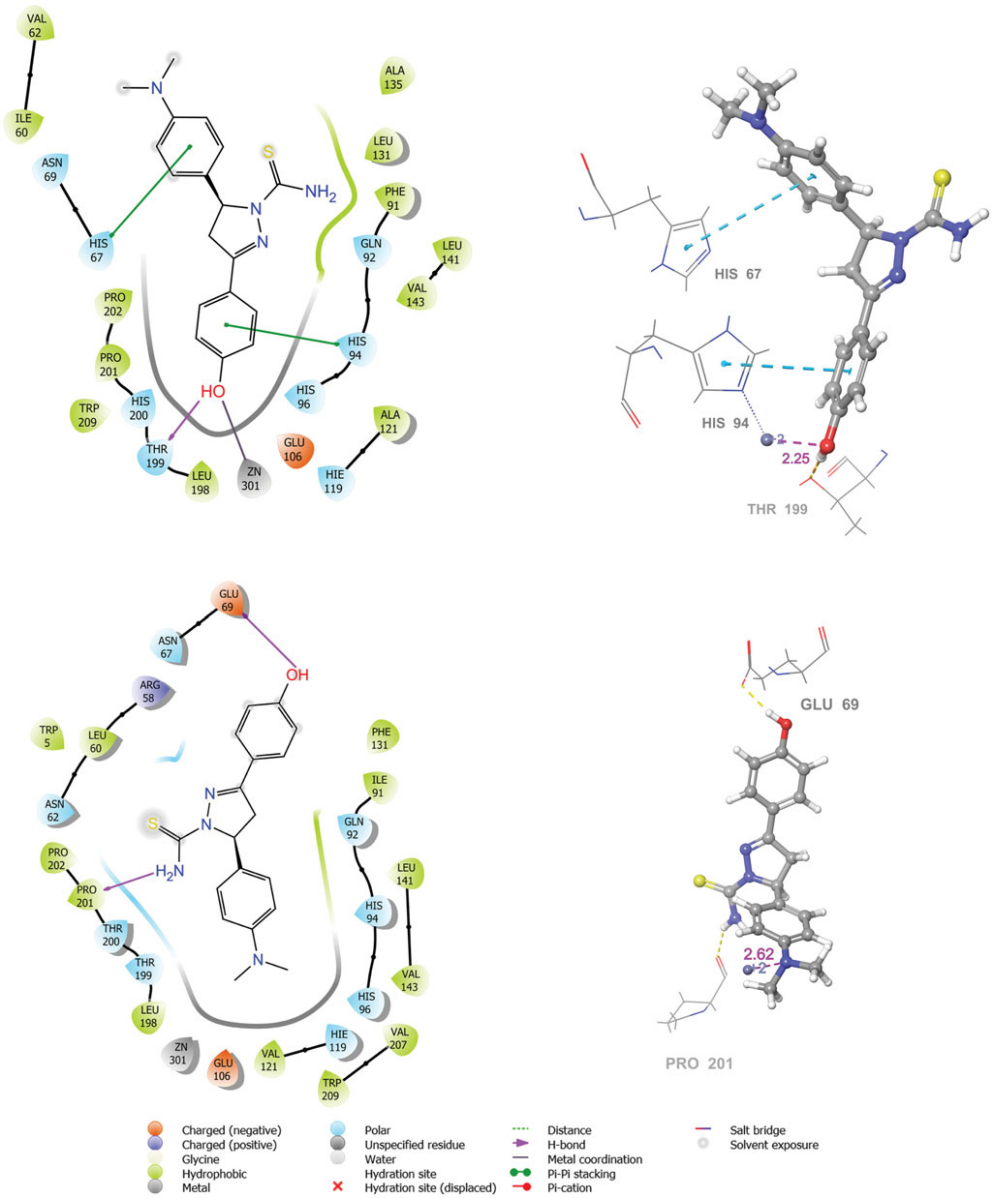
The 2 D and 3 D ligand interaction diagrams of hCA I-10 **(*S*)** (top) and hCA II-**10 (*S*)** (bottom) complexes obtained from prime MM-GBSA using Glide XP docked poses (In 3 D representation, hydrogen bonds are shown with yellow dashed lines, π-π interactions are shown with cyan colour. The distance between zinc metal and interacting atom is shown with pink colour).

The active site of both hCAI and II, as in all other CAs, is lined with both hydrophobic and hydrophilic residues (Figure 3). Hydrophobic part is believed to be responsible for entrapping the lipophilic CO_2_ molecule, and the other the part of the active site helps releasing the polar components after CO_2_ hydration reaction to the environment.^32^ All the synthesised new compounds were found to have low IC_50_ values in the low nanomolar range (Table 1). Although it is well known that the commonly used docking software available at the market performs well in predicting the active conformations of the biologically active compounds but the present scoring functions are not expected to discriminate between active and inactive compounds.^33^ We have obtained satisfying results using Glide in our recent studies.^34^ In the current study, in order to improve the docking scores, we performed more rigorous binding energy calculations using MM-GBSA protocol. We have let the residues in 3 Å distance from the ligands to be relaxed during the computations. MM-GBSA ΔG_binding_ values substantially agreed well with the experimental inhibition data. According to the MM-GBSA calculations, compound **6** scored top in both hCA isoforms. It is noteworthy to mention that, according to the IC_50_ results listed in Table 2, the compound **10** showed a slight hCA I versus hCA II selectivity, with a selectivity ratio (SR) of 3.46. The 2 D and 3 D ligand interaction diagrams of both compounds with the hCA I and II isoforms are shown in Figures 4 and 5. Compound **6** binds in a rather similar manner with the active site of both isoforms. The amino group of aniline part acts as a hydrogen bond donor and interacts with the TYR199 residue while the aromatic part establishes a π-π interaction with the residue HIS94. It also has favourable interactions with the polar residues at the active site entrance. Compound **10** has same type of interactions with the critical residues at the binding site of hCA I: phenolic -OH group participates in hydrogen bonding with THR199 and the same π-π interaction of the aromatic ring with the residue HIS94 is observed. Interestingly, the orientation is inverted in the active region of hCA II. The *N,N*-dimethyl aniline moiety is buried deep in the active site whereas phenol interacts with the negatively charged residue GLU69. The lack of contacts with the key residues HIS 94 and THR 199 could be responsible for the decreased activity towards hCA II.

**Table 2.** Glide XP Docking scores, MM-GBSA Δ*G*_binding_ energy values and selected molecular properties of compounds **1–10** and **AZA**.

Comp	Dscore (GlideXP)(kcal/mol)		MMGBSAdG Bind (kcal/mol)		Dipole μ (D)	QPlogPo/w^a^	%Human oral absorption^b^	PSA^c^	Rule of five
	hCA I	hCA II	hCA I	hCA II					
**1**	−4.59	−4.05	−33.89	−38.42	6.19	3.11	100.00	76.58	+
**2**	−4.89	−3.12	−33.83	−22.51	2.29	4.44	100.00	50.28	+
**3**	−4.12	−2.69	−35.34	−28.05	3.85	4.06	100.00	50.29	+
**4**	−4.35	−3.89	−35.19	−29.32	4.47	2.91	100.00	68.95	+
**5**	−4.58	−3.35	−34.16	−34.74	5.18	3.35	100.00	53.94	+
**6 (*R*)**	−4.31	−4.52	−47.95	−42.91	11.54	3.15	96.37	95.65	+
**7 (*S*)**	−4.51	−3.19	−37.08	−38.80	6.26	4.67	100.00	69.34	+
**8 (*S*)**	−4.45	−3.79	−38.70	−38.47	8.63	4.40	100.00	69.38	+
**9 (*S*)**	−4.00	−3.77	−44.31	−31.38	11.23	2.97	94.83	88.11	+
**10 (*S*)**	−4.13	−3.58	−42.48	−24.89	7.82	3.34	100.00	72.54	+
**AZA**	−5.93	−6.34	−29.97	−35.51	13.35	−1.77	44.26	133.03	+

^a^Logarithm of the partition coefficient of the compound between n-octanol and water (recommended value <5).

^b^Percentage of human oral absorption (<25% is weak and >80% is strong).

^c^Polar surface area (recommended value ≤140Å^2^).^35^

We have also calculated some molecular descriptors commonly used in absorption, distribution, metabolism and excretion (ADME) analysis (Table 2). As could be seen from the table, all of the new compounds obey Lipinski’s rule of five, which is an indication of the drug-likeness of a molecule, and PSA values are within the range that Veber et al. suggested.^35^

## 4. Conclusion

In the current study, starting from some chalcones, design, synthesis and characterisation of new pyrazole derivatives were reported. All the synthesised compounds were then evaluated for their inhibitory properties against hCA I and hCA II isoenzymes. They exhibited significant inhibitory features at low nanomolar concentrations ranging between 21.98 and 25.14 nM. Molecular docking studies further supported observed inhibitory profiles. Compound **10** which had slight hCA I versus hCA II selectivity, binds with hCA I in similar orientations with other compounds but it adopts different conformation in the active site of hCA II. According to the *in silico* molecular properties calculations, all compounds also obeyed the drug likeness properties. The new compounds presented in this study, might be promising lead compounds for the development of more selective and potent inhibitors as alternatives to the classical CA inhibitors.
